# Impact of Al^3+^ Concentration on Montmorillonite Sedimentation: Insights Into Particle Size Behavior

**DOI:** 10.1002/open.202500446

**Published:** 2025-12-03

**Authors:** Hanyu Zhao, Lijun Liu, Xue Yuan, Qingtao Pang, Lei Yao, Bo Gao, Yihong Li, Liang Han, Yizhe Yang, Wei Yu, Zhen Li, Yuexian Yu, Jinzhou Qu

**Affiliations:** ^1^ School of Chemistry and Chemical Engineering Xi’an University of Science and Technology Xi’an P. R. China; ^2^ Shanxi Coal Industry New Energy and Coal Preparation Technology Co. Ltd. Xi’an P. R. China

**Keywords:** coagulation, DLVO theory, montmorillonite, particle size, zeta potential

## Abstract

Particle size is crucial for sedimentation processes. However, there is limited research on how varying particle sizes influence the sedimentation of fine‐grained particles. This study examined the aggregation behavior of two types of montmorillonite particles with different sizes under different concentrations of Al^3+^. The findings indicated that the sample of fine particles exhibited a more rapid reduction in turbidity and a greater increase in the absolute value of surface potential across various Al^3+^ concentrations. Despite this, both samples achieved comparable final supernatant turbidity, sediment layer height, and residual particle size, suggesting that the enhancing effect of Al^3+^ on the sedimentation of fine‐grained montmorillonite is limited by particle size. Different concentrations of Al^3+^ result in the formation of hydroxyl compounds and hydroxides that adsorb onto mineral surfaces, promoting the sedimentation of finer‐grained montmorillonite in the sample of fine particles. DLVO theory confirmed that the electrical double layers of the samples of coarse and fine particles gradually diminished under the influence of Al^3+^, effectively enhancing the aggregation of montmorillonite. Furthermore, thermodynamic analysis suggested that particles smaller than 500 nm do not settle further, even with the addition of aluminum ions.

## Graphical abstract

The current research essentially focuses on the coagulation and sedimentation characteristics of particles with two distinct particle‐size compositions. The DLVO theory confirms that under the influence of aluminum ions, the coarse and fine particle sampleS of montmorillonite essentially possess the effect of eliminating or weakening the “barrier”.

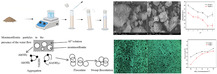

## 1 Introduction

Clay minerals such as kaolinite and montmorillonite serve as essential industrial materials, finding extensive applications in ceramics manufacturing, paper production, cosmetic fillers, ink additives, and dental care products. However, in mineral processing systems, such minerals often persist as detrimental impurities. Montmorillonite, with its distinctive layered physicochemical structure, poses significant challenges to flotation processes and coal slurry water treatment, particularly in coal production, where its dispersibility and hydration‐swelling properties exacerbate processing complexities.

The difficulties in coal slurry water treatment primarily stem from its mineral composition characteristics. Studies indicate that clay minerals and quartz collectively account for over 60% of the total mineral mass in coal slurry water, with clay particles typically measuring below 2 μm and exhibiting pronounced mud‐forming tendencies [1]. These negatively charged particles maintain colloidal stability through electrostatic repulsion, while the coexistence of multiple particle‐size fractions in recirculated water systems further impedes sedimentation. Additionally, the strong hydrophilicity of clay minerals promotes hydration membrane formation on particle surfaces, hindering reagent adsorption and particle aggregation [2, 3]. Notably, montmorillonite has adverse effects on sedimentation due to its high electronegativity, significant hydration expansion capacity, and enhanced dispersibility. While existing research predominantly focuses on ultrafine particles (<2 μm), the continuous water recycling in coal preparation plants may generate diverse particle‐size distributions of montmorillonite. Nevertheless, systematic investigations into sedimentation differences among micron‐sized montmorillonite particles with varying size compositions remain scarce.

To mitigate the inefficient sedimentation of fine montmorillonite particles, researchers have investigated metal ion‐induced aggregation techniques. Yang [4] demonstrated that Ca^2+^ and Al^3+^ ions exhibit concentration‐dependent improvements in montmorillonite sedimentation, with supernatant turbidity decreasing significantly as ion valence and concentration increase. Liu [5] further revealed that high‐valence ions (e.g., Al^3+^) enhance electrical double‐layer compression and surface adsorption through their elevated charge density and smaller ionic radii, thereby suppressing montmorillonite swelling capacity and promoting agglomeration. These phenomena correlate closely with particle charge density characteristics. Early studies by Jonas and Robertson established an inverse relationship between clay particle size and charge density [6], subsequently validated by JAN [7] through bentonite experiments. However, contradictory findings emerge from Shi [8] and Yamanaka [9], the former observed decreasing surface charge density with increasing particle size in nanosilica. At the same time, the latter reported elevated effective charge density in smaller SiO_2_ particles. This discrepancy suggests potential system‐dependent mechanisms governing particle size effects on charge density, which may critically influence electrolyte‐mediated sedimentation regulation. Yang [10] further confirmed strong correlations between metal ion adsorption efficiency and particle surface charge density. Building on these findings, this study systematically examines the influence of particle size on coagulation‐sedimentation by analyzing two distinct size fractions of fine montmorillonite particles across varying Al^3+^ concentrations.

Through comprehensive analyses of turbidity, zeta potential, and residual particle size distribution, we elucidate the flocculation mechanism variations across different particle size classes, aiming to advance the understanding of clay mineral adsorption–sedimentation mechanisms from a particle size perspective.

## 2 Methods

### 2.1 Sample

The montmorillonite samples of various particle sizes used in the experiment, with montmorillonite purity greater than 90%, were purchased from Anhui Junhong New Materials Co., Ltd. in China.

### 2.2 Test Methods

#### 2.2.1 Suspension Preparation

A well‐dispersed montmorillonite suspension (10 g/L) was prepared by adding 2.5 g of montmorillonite powder (varying particle sizes) to a 500 mL beaker containing 200 mL of deionized water. The mixture was agitated vigorously using a magnetic stirrer for 30 min to ensure homogenization. The suspension was immediately transferred to a 250 mL stoppered graduated cylinder and adjusted to a final volume of 250 mL.

#### 2.2.2 Natural Sedimentation Protocol

For natural sedimentation, the montmorillonite suspension (250 mL) was inverted 10 times in a stoppered graduated cylinder and allowed to settle undisturbed for 1 h. The recession of the clarification interface was tracked throughout the sedimentation process. After settling, the sediment layer height was measured, and 20 mL of the upper supernatant was aspirated using a pipette. Turbidity was analyzed.

#### 2.2.3 Cohesive Sedimentation with Al^3+^ Additives

**FIGURE 1:**
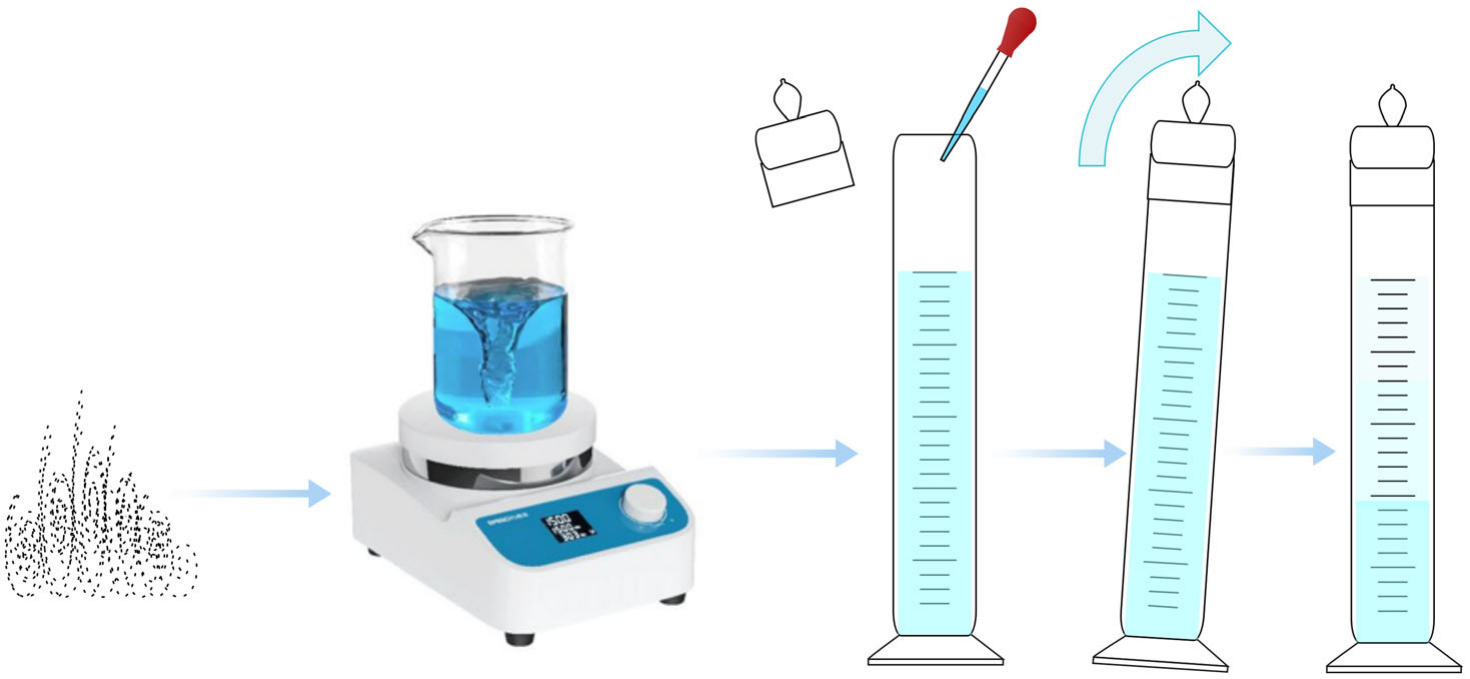
Schematic representation of the montmorillonite sedimentation process.

To evaluate cohesive sedimentation, 240 mL of the prepared montmorillonite suspension was combined with 10 mL of Al^3+^ solutions (5–30 mmol/L) in a graduated cylinder. The mixture was inverted 10 times to ensure thorough mixing and allowed to settle for 1 h. Then the recession of the clarification interface, the turbidity, and the residual particle‐size distribution were measured. A schematic of the sedimentation process is illustrated in Figure 1.

### 2.3 Analytical Methods

#### 2.3.1 Sedimentation and Turbidity Analysis

Sedimentation efficiency was quantified by measuring the sediment layer height (after 1 h) and supernatant turbidity (analyzed via WZS‐188 turbidimeter). Lower turbidity values and increased sediment compaction indicated enhanced settling performance.

#### 2.3.2 Zeta Potential and Particle Size Analysis

The surface zeta potential of supernatant samples was measured using a ZEN3690 analyzer (Malvern, UK). Three replicate measurements were conducted per sample, with results averaged to ensure reproducibility. Residual particle‐size distribution in the supernatant was subsequently quantified using the same analytical instrument.

## 3 Experimental Results and Discussion

### 3.1 Sample Properties

**FIGURE 2:**
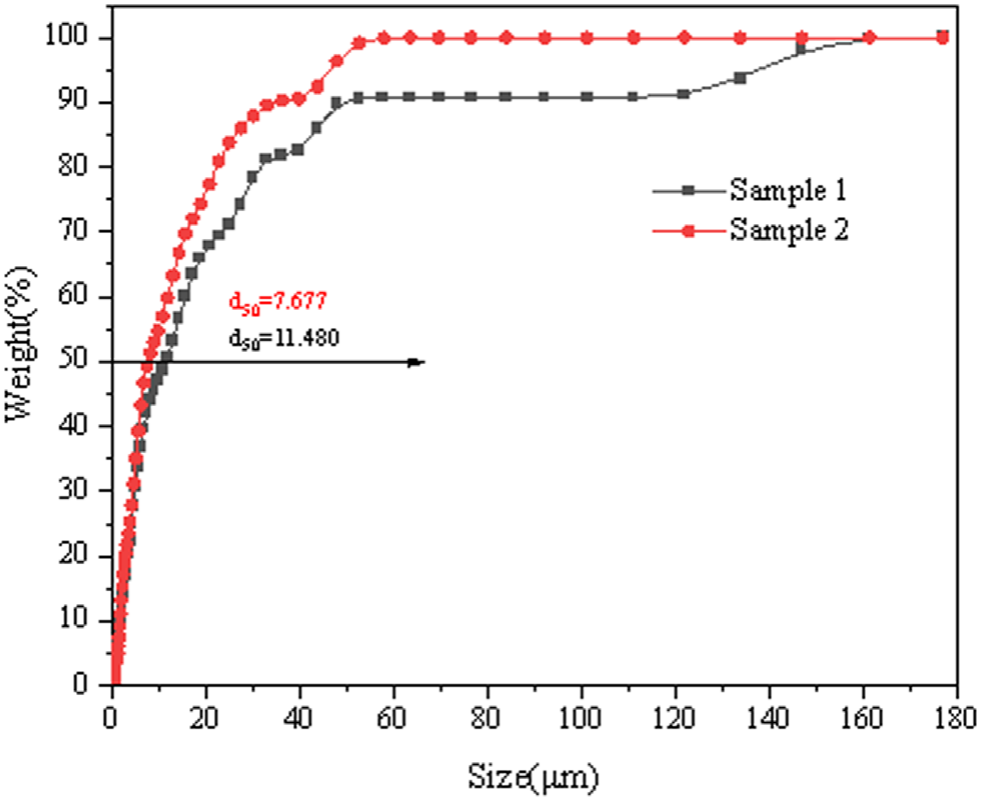
Particle size analysis of montmorillonite samples.

**FIGURE 3:**
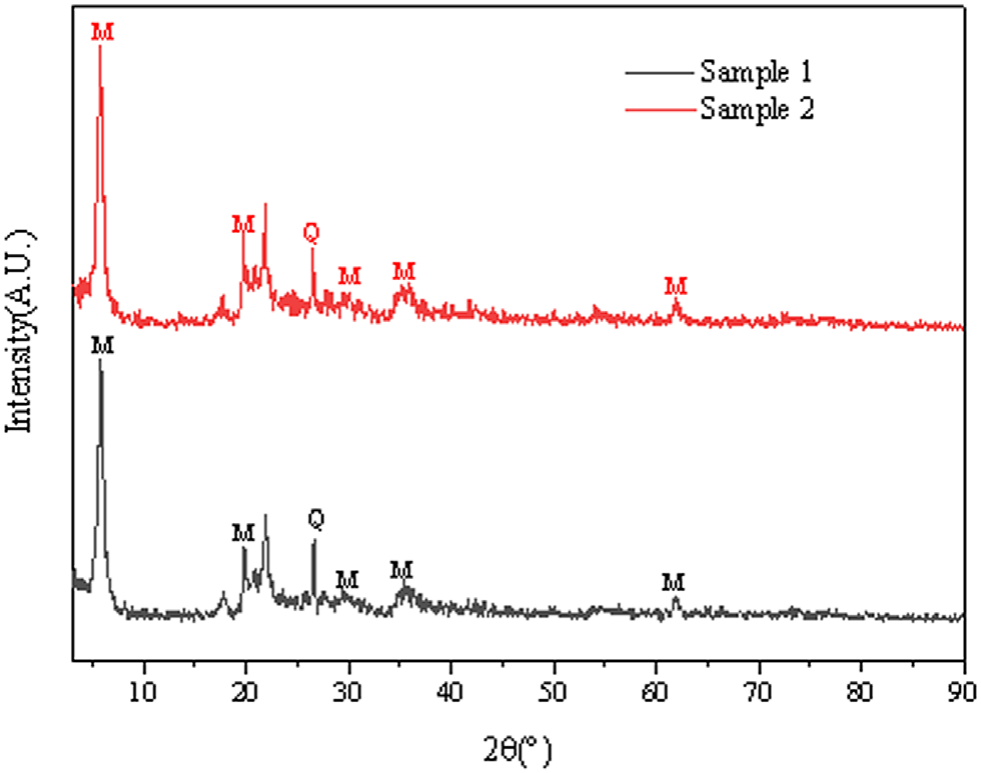
X‐ray diffraction analysis of montmorillonite samples.

**FIGURE 4:**
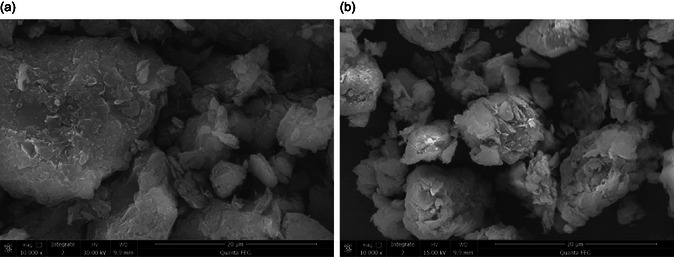
SEM of montmorillonite samples. (a) Sample 1; and (b) Sample 2.

**FIGURE 5:**
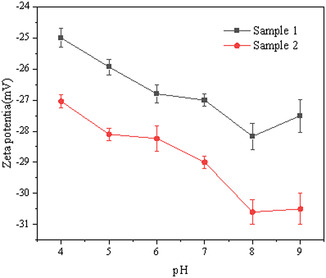
Zeta potential analysis of montmorillonite samples.

**FIGURE 6:**
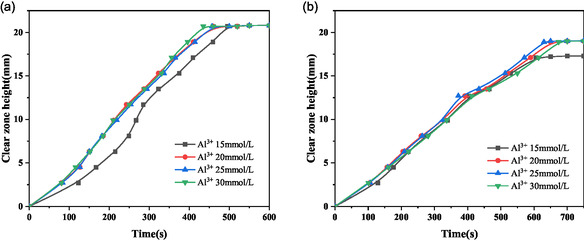
Evolution of the settling height for the understudied montmorillonite samples. (a) Sample 1; and (b) Sample 2.

To investigate the settling behavior of montmorillonite across varying particle sizes, the results of the analysis of particle‐size composition, material composition, surface structure, and surface potential of the samples are illustrated in Figures 2–6.

#### 3.1.1 Particle Size Analysiss

As illustrated in Figure 2, Sample 1 demonstrates a broad particle‐size distribution spanning from 1.87 to 143.7 μm, with a median particle size (d_50_) of 11.48 μm and a d_90_ value of 46.51 μm. Throughout this study, we designate this as the coarse particle sample. In contrast, Sample 2 exhibits a narrower distribution ranging from 1.736 to 48.98 μm, featuring a refined d_50_ measurement of 7.67 μm and a corresponding d_90_ of 35.08 μm. This distinctively finer material will hereafter be identified as the fine particle sample in subsequent analyses.

#### 3.1.2 Mineral Composition

According to the plotted results in Figure 3, the mineral composition test and analysis show that the crystal structures of the main mineral montmorillonite are basically the same. From the X‐ray diffraction analysis results, the montmorillonite has a relatively high purity and contains a small amount of quartz, which has little interference with the adsorption process of Al^3+^ on montmorillonite. The basal spacing (d_001_) measured along the (001) crystallographic plane of montmorillonite was determined to be 1.36 nm, which conclusively identifies the sample as a calcium‐based montmorillonite structure.

### 3.2 Specific Surface Area (SSA)

Based on the results shown in Table 1, Sample 1 and Sample 2 exhibit different SSA. Among them, the SSA of Sample 2 is larger, at 52.9531 m^2^/g, while that of Sample 1 is smaller, at 48.5517 m^2^/g. A larger SSA allows the samples to adsorb more cations, resulting in an increased thickness of the diffuse layer. According to the electric double layer theory [11], the SSA is positively correlated with the thickness of the electric double layer. Therefore, the electric double layer of Sample 2 is thicker, leading to stronger electrostatic repulsion between particles and greater resistance to sedimentation.

**TABLE 1:** BET surface area of montmorillonite samples.

	Specific surface area (m^2^/g)
The sample of coarse particles	48.5517
The sample of fine particles	52.9531

### 3.3 SEM

As shown in Figure 4, the surfaces of the montmorillonite samples are relatively rough and uneven. A comparison of the surface structures of Sample 1 and Sample 2 indicates that Sample 1 consists of thicker particles with a compact structure. Although stacked lamellar structures can be observed on its particle surfaces, the overall undulation is minor, and the surface appears relatively flat and smooth, corresponding to a smaller SSA. In contrast, Sample 2 contains smaller and more loosely distributed particles; its surface consists of irregularly stacked lamellae, which make it rougher and expose a larger surface area, thereby resulting in a higher SSA.

#### 3.3.1 Zeta Potential

From Figure 5, the zeta potential values of montmorillonite are all negative in the pH range of 4–9, and the absolute value of the zeta potential rises with the pH growth. In addition, the absolute value of the zeta potential of the fine particles is larger than that of the coarse particles. The charge distribution density on the surface of the fine particles is high, which can attract ions with opposite charges more strongly to form an electric double layer. The ion distribution in its compact layer and diffuse layer is relatively denser. The denser ion distribution will have a more remarkable neutralizing effect and potential change on the surface charge of the fine particles, resulting in a relatively larger absolute value of the surface potential of the fine particles. This will enhance the stability of the montmorillonite solution and make it more difficult for montmorillonite particles to aggregate and settle.

### 3.4 Natural Sedimentation

Before the flocculation test, a natural sedimentation test was conducted. The obtained results revealed that the sedimentation effect of Samples 1 and 2 under natural conditions was weak, with turbidity of 2378 NTU in the supernatant of Sample 1 and 4970 NTU in the supernatant of Sample 2.

### 3.5 Analysis of Sedimentation Effect of Both Coarse and Fine Montmorillonite Samples in the Presence of Various Al^3+^ Ions

**FIGURE 7:**
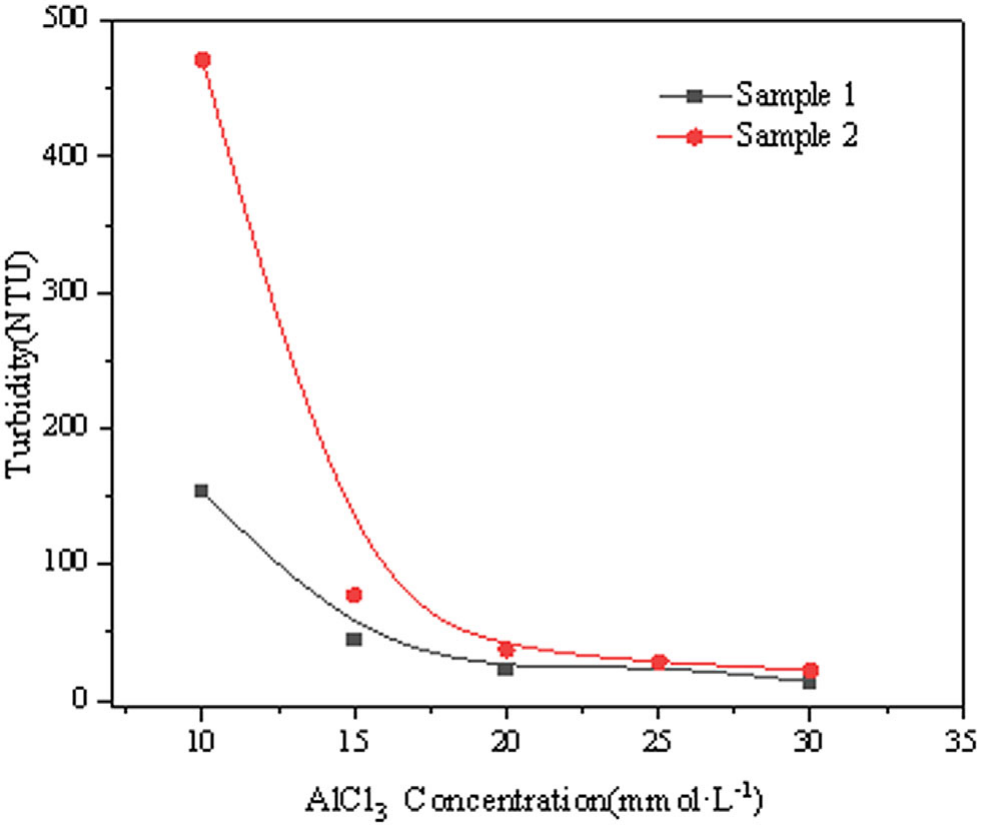
Supernatant turbidity in terms of the AlCl_3_ concentration for both samples.

**FIGURE 8:**
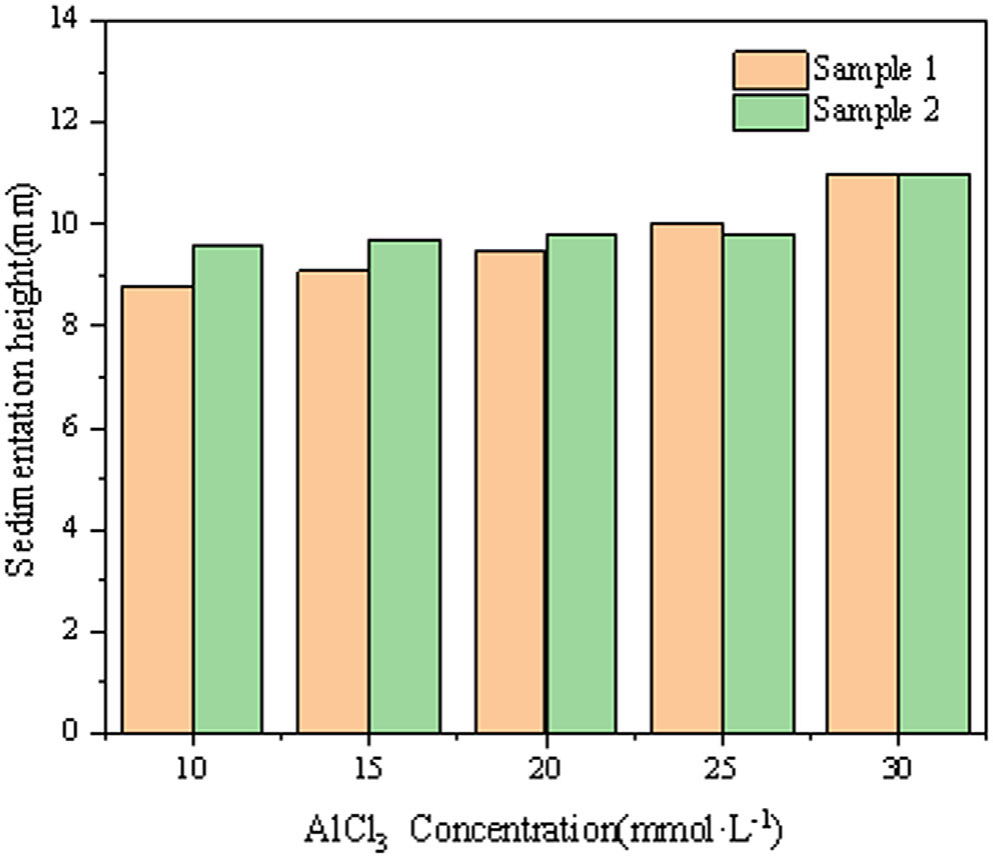
Sediment layer heights of both samples for various concentrations of AlCl_3_.

This subsection methodically examines the influence of Al^3+^ concentration on the sedimentation behavior of montmorillonite particles of varying sizes. The sedimentation rate is detailed in Figure 6, the turbidity of the supernatant is quantified in Figure 7, and the evolution of the sediment layer height is illustrated in Figure 8.

It can be seen from Figure 6 that under the same sedimentation conditions, with the increase of ion concentration, when coarse and fine particles are added with metal cations for aggregation and sedimentation, the sedimentation rate of the formed flocs does not grow significantly. Comparing the total sedimentation time of coarse and fine particle samples at the same ion concentration, the sedimentation time of coarse particles is shorter than that of fine particle samples. The sedimentation process for coarse particles concludes within 500 s, whereas that for fine particles require 700 s to reach completion. Therefore, it is observed that the concentration of metal cations does not substantially alter the size of the floc structure formed during the montmorillonite sedimentation process, and consequently, there is no significant variation in the sedimentation rate of coarse and fine samples with increasing ion concentration. Therefore, when metal cations are added, the changes in turbidity and height of the sedimentation layer during the sedimentation process of montmorillonite with different particle sizes are more related to the number of deposited montmorillonite particles. Therefore, when metal cations are added to adjust the montmorillonite sedimentation process, the sedimentation effect would be mainly related to the particle size of the original particles and the concentration of minerals. Particle properties also play a significant role in influencing sedimentation behavior. Qiu et al. [12] found that with the growth of the layer charge density, the surface area of the particles increases. In contrast, the absolute values of the zeta potential, the colloid value, and the swelling amount all exhibit a descending trend, and the dispersibility is also reduced.

As shown in Figures 6–, 8, the addition of metal ions significantly enhances the sedimentation of montmorillonite particles. Increasing ion concentration correlates with a steady decline in supernatant turbidity, suggesting progressive particle aggregation and settling. Beyond 20 mmol/L ion concentration, turbidity values stabilize, indicating saturation of the flocculation effect. At 30 mmol/L, both coarse and fine particle samples achieve minimal turbidity, reflecting near‐complete sedimentation and the lowest residual solid content in the supernatant.

Sediment layer height increases with higher ion concentrations for both particle types, as elevated Al^3+^ levels promote the formation of larger, more numerous flocs. At low ion concentrations, fine particles exhibit a comparatively greater sediment height, likely due to their smaller size and higher particle count per unit mass, which generates numerous small, loosely structured flocs. At high ion concentrations (>20 mmol/L), the sediment heights of coarse and fine particles converge, reaching a maximum of 11 mm at 30 mmol/L. This uniformity implies that sufficient Al^3+^ eliminates size‐dependent differences in floc density and compaction.

### 3.6 Surface Potential

The effect of various concentrations of Al^3+^ ions on the zeta potential of montmorillonite at two particle sizes was measured by zeta potential and nanoparticle size analyzer, as illustrated in Figure 10.

**FIGURE 9:**
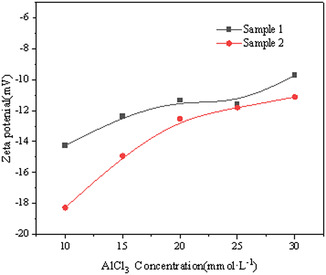
Effect of the Al^3+^ concentrations on the zeta potential of the understudied montmorillonite samples.

As illustrated in Figure 9, both Sample 1 and Sample 2 exhibit a progressive decline in zeta potential values as ion concentration increases, with Sample 2 displaying significantly larger absolute zeta potential variations compared to Sample 1 at equivalent concentrations. Sample 2 exhibits high surface atomic coordination unsaturation and numerous surface‐active sites, likely enhancing their adsorption affinity for aluminum ions. This preferential adsorption alters surface charge distribution, resulting in a pronounced zeta potential shift. In contrast, coarse particles, with fewer surface‐active sites, adsorb fewer aluminum ions, leading to a smaller zeta potential variation.

As the surface area grows with the lessening of the particle size, the adsorption rate of Al^3+^ ions on the surface of Sample 2 increases slightly, effectively compressing the surface double layer, leading to a remarkable reduction of the zeta potential of Sample 2. In addition, Sample 2 possesses a small particle size and a large number of particles, which leads to high turbidity of the supernatant and a lower overall sedimentation effect compared to Sample 1, resulting in a lower potential than Sample 1.

### 3.7 Size Analysis of Floc Size

**FIGURE 10:**
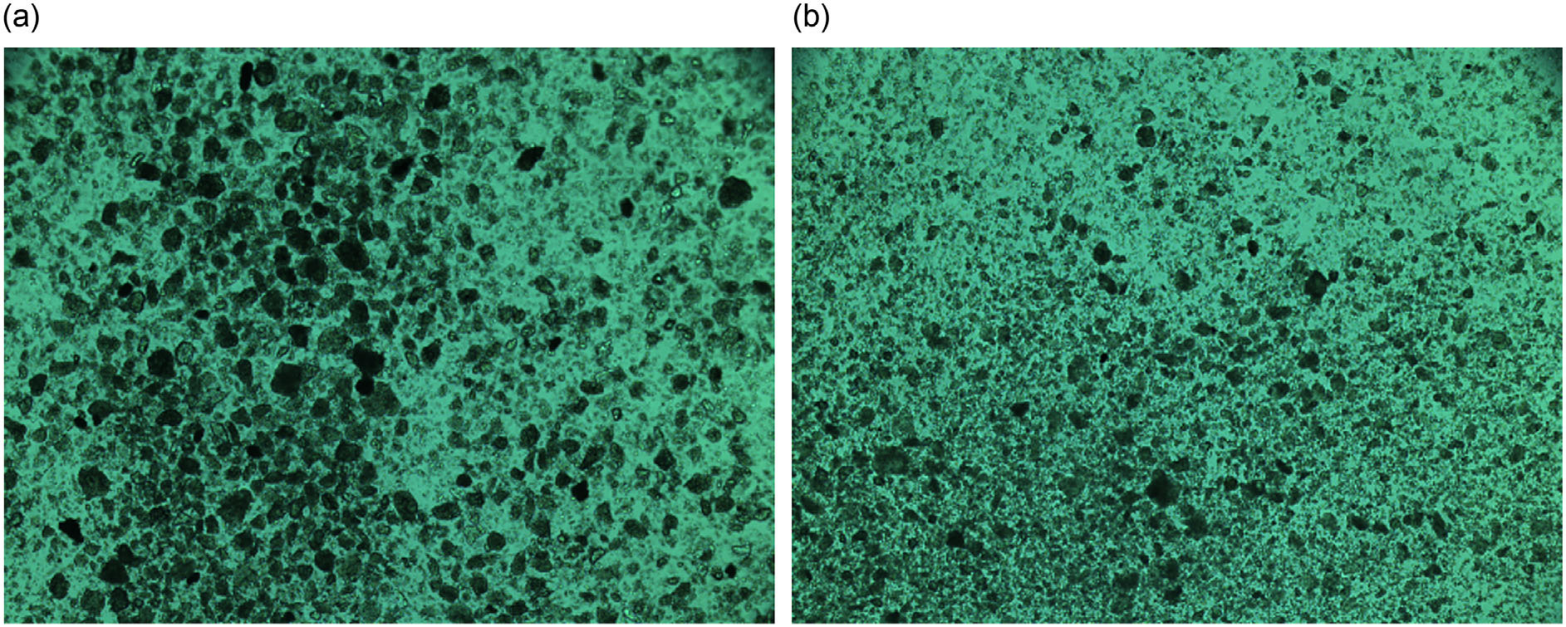
Micromorphology of flocs in the understudied montmorillonite samples under the action of Al^3+^ ions. (a) Sample 1; and (b) Sample 2.

When a 30 mmol/L Al^3+^ solution was added to montmorillonite suspensions with different particle sizes, the suspensions were allowed to settle. After clarification of the supernatants, the flocs were aspirated using a rubber‐tipped dropper and placed on glass slides. The slides were then examined under an optical microscope to observe the flocculation morphology, and the results are shown in Figure 10.

**FIGURE 11:**
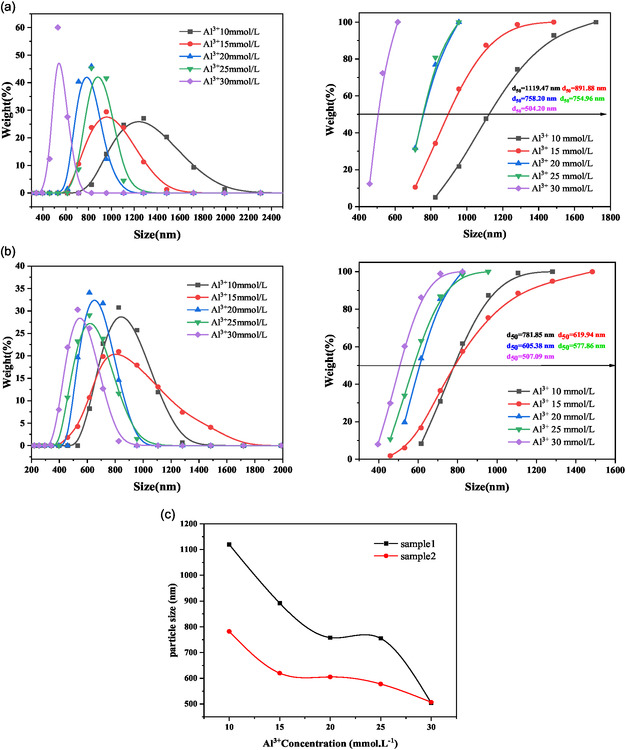
Particle size distribution of the supernatant for both samples after sedimentation of montmorillonite: (a) Sample 1; (b) Sample 2; and (c) plots of the particle size in terms of the concentration.

The size and compactness of flocs directly influence the settling efficiency. When flocs are small and loose, the settling rate is slow and the sedimentation efficiency is poor. Conversely, large and compact flocs settle rapidly, resulting in significantly improved efficiency [13]. As shown in Figure 11, after adding the 30 mmol/L Al^3+^ solution, both Sample 1 and Sample 2 exhibited aggregation into clusters, but clear differences were observed in their floc morphologies. The flocs in Sample 1 were larger and more closely connected, resulting in a faster settling rate. In contrast, the number of large flocs in Sample 2 was markedly lower, and the overall compactness decreased, leading to slower sedimentation.

In Sample 1, the particles possess a smaller SSA and a thinner electric double layer, which leads to stronger compression of the double layer. This reduces the electrostatic repulsion between particles, facilitating the formation of dense flocs. Moreover, the larger particle size increases the likelihood of collisions during sedimentation, further promoting the formation of large and compact flocs. Conversely, the smaller and more numerous particles in Sample 2 tend to form small flocs. Although the hydrolysis products of aluminum create a network through a bridging effect, the resulting structure is loose and retains a considerable amount of water. Consequently, the flocs are prone to dispersion and breakage during sedimentation, remaining loose and leading to a slower settling rate and lower sedimentation efficiency.

### 3.8 Trends in Particle Size Changes of Supernatant

Following Al^3+^ enhanced coagulation, residual fine particles persisted in the supernatant of both coarse and fine montmorillonite suspensions. Laser diffraction particle size analysis in Figure 11 revealed distinct size distribution profiles for these residual particles under Al^3+^ treatment.

With increasing ion concentration, the particle size in the supernatant decreases for both samples. The average particle size of Sample 1 declines progressively, reaching a minimum of 504.2 nm at 30 mmol/L ion concentration 35. Similarly, Sample 2 demonstrates a reduction in average particle size, achieving 507.09 nm under the same ion concentration. As ion concentration rises, the residual particle sizes of both montmorillonite types in the supernatant gradually converge.

### 3.9 Analysis of the Principle of Aggregation and Sedimentation

The hydrolysis behavior of Al^3+^ ions in montmorillonite suspensions is strongly pH‐dependent. With varying pH, both the degree of Al^3+^ hydrolysis and the dominant species of hydrolysis products differ significantly. Optimal particle sedimentation occurs within a pH range of 6–8, where dominant hydrolysis products like Al(OH)_3_ form through controlled hydrolysis. Deviations from this range (pH < 6 or > 8) lead to altered hydrolysis pathways, generating less effective species that diminish sedimentation efficiency.

When AlCl_3_ is under the condition of pH = 7, Al^3+^ mainly undergoes hydrolysis reactions, and its existing forms are relatively complex, mainly in the following forms: Al^3+^ + 3H_2_O ⇌ Al(OH)_3_ + 3H^+^. Al^3+^ undergoes hydrolysis reactions in an aqueous solution, and the hydrolysis equation is as follows: Al^3+^ + H_2_O ⇌ [Al(OH)]^2+^ + H^+^, [Al(OH)]^2+^ + H_2_O ⇌ [Al(OH)_2_]^+^ + H^+^. Under the condition of pH = 7, the concentrations of these hydrolysis product ions are very low. Theoretically, there will also be a tiny amount of free Al^3+^ that has not undergone hydrolysis in the solution.

The adsorption‐bridging effect of Al(OH)_3_ aggregates dispersed coal slime particles into larger flocs by connecting multiple particles through its linear or branched polymeric structure. As floc size increases, gravitational forces acting on the flocs overcome buoyancy and hydrodynamic resistance, enabling accelerated sedimentation. The abundant active surface sites on Al(OH)_3_ facilitate multidentate interactions with particle surface functional groups via hydrogen bonding and van der Waals forces, enhancing particle‐bridging efficiency. This mechanism effectively counteracts the colloidal stability of finely dispersed coal slime particles in aqueous systems.

**FIGURE 12:**
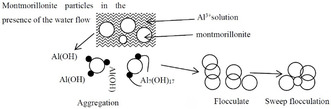
Mechanism of aggregation and sedimentation by Al^3+^.

The formation of Al(OH)_3_ precipitates creates a three‐dimensional network structure in solution that encapsulates and entraps adjacent coal slime particles during sedimentation. As illustrated in Figure 12, These precipitates occupy spatial domains within the aqueous medium, generating hydrodynamic drag forces that mobilize and entrain surrounding colloidal particles as they descend. Fine coal slime particles, originally stabilized by colloidal forces, become aggregated through physical entrapment within the Al(OH)_3_ matrix and cosediment rapidly under enhanced gravitational forces. This sweep‐flocculation mechanism significantly accelerates the settling velocity of suspended particles in slime water systems by effectively overcoming their inherent colloidal stability. The aggregation–sedimentation principle aligns with established interfacial interaction dynamics in particle‐laden fluid systems.

As the ion concentration grows, it can be seen from Figure 9 that the absolute value of the surface potential of particles gradually lessens and the electrostatic repulsion reduces. However, many factors affect this data, so it cannot be utilized as a criterion to judge the aggregation process. Therefore, the reciprocal of the Debye constant, that is, the thickness of the diffuse layer ($\kappa^{-1}$) is adopted as an appropriate criterion. For an aqueous solution at 25°C, $\kappa =3.29\times 10^{9}{\left(\sum c_{i}Z_{i}^{2}\right)}^{1/2}m^{-1}$ (where *κ* represents the Debye constant, and ci and Zi in order are the amount‐of‐substance concentration and the charge number of ions). According to this formula, as the ion concentration rises, the value of κ increases and thereby the thickness of the diffuse layer ($\kappa^{-1}$) will become smaller. Based on the added concentration of Al^3+^, the calculated thicknesses of its diffuse layer are presented in Table 2.

**TABLE 2:** The thickness of the diffuse layer at various Al^3+^ concentrations ($\kappa^{-1}$/nm).

Al^3+^ Concentration (mol/L)	2 × 10^−4^	4 × 10^−4^	6 × 10^−4^	8 × 10^−4^	10 × 10^−4^	12 × 10^−4^
$\kappa^{-1}$/nm	7.16	5.06	4.14	3.58	3.20	2.92

This formula demonstrates that increasing ionic strength elevates the Debye parameter (κ), inversely reducing the diffuse layer thickness ($\kappa^{-1}$). Calculated ($\kappa^{-1}$) values for Al^3+^ across tested concentrations quantitatively confirm this inverse relationship, as systematically tabulated in Table

As the concentration of Al^3+^ increases, the thickness of the diffuse layer between particles gradually lessens, the intermolecular attractions among particles grow, and the particle aggregation process can be carried out more quickly. However, this does not reflect the differences in the sedimentation process between the two different types of particles.

### 3.10 Thermodynamic Analysis of Floc Separation

Evaluating entropy variations in solid–liquid floc separation necessitate employing idealized thermodynamic frameworks to isolate dominant energy exchange pathways. These assumptions will help describe the thermodynamic equations relevant to particle solid–liquid separation systems in the context of floc separation. In solid–liquid flocculation systems, thermodynamic parameters like entropy and free energy establish quantitative criteria for assessing the thermodynamic viability of floc separation processes. The separation criterion fundamentally derives from the energy balance between interfacial interactions and bulk phase reorganization [14]:

(1) $$K=\frac{\pi g\text{Δ}\rho d^{3}H\left(1-\phi_{F}\right)}{12kT\ln\left(1/\phi_{F}\right)}$$

Where Δρ denotes solid–liquid density difference: $\Delta \rho =\rho_{S}-\rho_{L}$, *g* represents gravitational acceleration; *H* is the height of the floc suspension before settling; *T* denotes the absolute temperature, k=R/L is the Boltzmann constant, $k=1.38\times 10^{-23}$ J/K.

The separation criterion magnitude is governed by two primary factors: settling height (*H*) and particle diameter (*d*). Experimental parameters for montmorillonite flocculation‐sedimentation were maintained at a slurry temperature of 20°C through thermostatic control.

Let us assume that the density of montmorillonite is ≈2 g/cm^3^, the volume concentration of flocs is 0.5%, Δρ = 1000 kg/m^3^, and the sedimentation height is 20 cm. In our experiment, particles with a diameter of 150 μm to 1 μm are aimed to be separated based on the laser particle size analysis results, and subsequently, the separation criterion can be calculated.

Given *g* = 9.80 m/s^2^, *k* = 1.38 × 10^−23^ J/K, $\phi_{F}=0.005$, and *H* = 0.02m, these parameters are substituted into Equation (1), where $(1-\phi_{F})/(\ln(1/\phi_{F})=0.188$. For particle diameter *d* = 1.5 × 10^−4^ m, the permeability coefficient evaluates to *K* = 8.04 × 10^9^. When considering finer particles with *d* = 1 μm (*d* = 1 × 10^−6 ^m), the corresponding permeability decreases significantly to *K* = 2.38 × 10^3^.

Thermodynamic analysis confirms that at a feed floc volumetric concentration of 0.5%, even with particle diameter *d* = 1 μm, the separation criterion (*K*) significantly exceeds unity, demonstrating that flocculation separation can theoretically proceed spontaneously. When the particle size decreases to 750 nm, *K* ≈ 1 marks the critical threshold for sedimentation feasibility. For particles below 750 nm, spontaneous separation becomes thermodynamically unachievable, a conclusion aligning fundamentally with Stokesian free‐settling particle size limits in coal slurries [15], thus defining 750 nm as the natural sedimentation critical size.

Experimentally, untreated montmorillonite suspensions exhibited high turbidity, particularly in fine‐particle systems (<750 nm), confirming poor spontaneous settling. Post‐Al^3+^ addition, coarse‐particle suspensions showed progressive reduction in supernatant residual particle size (stabilizing at 504 nm with increasing Al^3+^ dosage), while fine‐particle suspensions reached 507 nm at maximum Al^3+^ dosage, converging toward comparable values. This indicates that Al^3+^ enhances floc sedimentation efficiency but is constrained by particle size limits: below 500 nm, Al^3+^ fails to enable effective settling.

Mechanistically, montmorillonite particles between 750 and 500 nm adsorb sufficient metal ions to promote aggregation and settling. Below 500 nm, ion adsorption plateaus, preventing further floc growth and rendering sedimentation kinetically unfavorable.

## 4 Conclusion

(1) The montmorillonite particle‐size distributions exhibit distinct differences: coarse particles range from 1.87 to 143.7 μm (d_50_ = 11.48 μm, d_90_ = 46.51 μm), while fine particles span 1.736–48.98 μm (d_50_ = 7.67 μm, d_90_ = 35.08 μm). Notably, both particle types maintain a near‐constant zeta potential difference of ∼3 mV, with fine particles displaying substantially higher absolute zeta potential values. This disparity confirms significantly enhanced surface charge density in fine particles compared to coarse particles, establishing dual potential gradients. Consequently, fine‐particle suspensions demonstrate superior colloidal stability under equivalent conditions.

(2) Post‐Al^3+^ addition, coarse montmorillonite particles retain marginally higher settling velocities than fine particles, achieving faster sedimentation completion. Supernatant analysis reveals distinct residual particle characteristics between coarse and fine fractions postsedimentation. At low Al^3+^ dosages, coarse‐particle systems maintain larger residual particle sizes in the supernatant. With increasing Al^3+^ concentrations, residual particle sizes converge across both particle classes, stabilizing near 500 nm. This demonstrates Al^3+^'s capacity to overcome inherent particle‐size‐dependent sedimentation thresholds in montmorillonite systems. The findings establish a critical theoretical framework for optimizing coal slime water treatment processes challenged by montmorillonite colloidal stability.

(3) Based on thermodynamic modeling of solid–liquid separation dynamics, particles exceeding 0.75 μm in montmorillonite suspensions achieve natural settling at 0.5% volumetric concentration. The critical sedimentation threshold for polydisperse systems is reduced to approximately 500 nm through Al^3+^ ion incorporation, attributed to enhanced interparticle aggregation and density modulation. However, sub‐500 nm particles exhibit persistent colloidal stability even with Al^3+^ supplementation, demonstrating size‐dependent limitations in overcoming Brownian motion and surface charge repulsion.

(4) Current research predominantly examines coagulation and sedimentation behaviors in systems with two particle‐size distributions. However, mechanistic interactions between multicomponent particle size fractions and their synergistic effects on flocculation dynamics remain unexplored, particularly regarding hierarchical aggregation patterns. Furthermore, a fundamental understanding of metal ion adsorption thermodynamics requires enhanced molecular‐level characterization of particle surface energetics and interparticle force modulation.

## Supporting Information

Additional supporting information can be found online in the Supporting Information Section. **Supporting Fig. S1:** Schematic representation of the montmorillonite sedimentation process. **Supporting Fig. S2:** Particle size analysis of montmorillonite samples. **Supporting Fig. S3:** X‐ray diffraction analysis of montmorillonite samples. **Supporting Fig. S4:** SEM of montmorillonite samples. (a) Sample 1; (b) Sample 2. **Supporting Fig. S5:** Zeta potential analysis of montmorillonite samples. **Supporting Fig. S6:** Evolution of the settling height for the understudied montmorillonite samples. (a) Sample 1; (b) Sample 2. **Supporting Fig. S7:** Supernatant turbidity in terms of the AlCl_3_ concentration for both samples. **Supporting Fig. S8:** Sediment layer heights of both samples for various concentrations of AlCl_3_. **Supporting Fig. S9:** Effect of the Al^3+^ concentrations on the zeta potential of the understudied montmorillonite samples. **Supporting Fig. S10**: Micromorphology of flocs in the understudied montmorillonite samples under the action of Al^3+^ ions. (a) Sample 1; (b) Sample 2. **Supporting Fig. S11:** Particle size distribution of the supernatant for both samples after sedimentation of montmorillonite: (a) Sample 1; (b) Sample 2; (c) Plots of the particle size in terms of the concentration. **Supporting Fig. S12**: Mechanism of aggregation and sedimentation by Al^3+^. **Supporting Fig. S13:** XPS spectra of montmorillonite at various Al^3+^ concentrations: (a) Pristine; (b) After sedimentation. **Supporting Fig. S14:** FTIR spectra of montmorillonite at various Al^3+^ concentrations. (a) Sample 1; (b) Sample 2. **Supporting Table S1:** BET Surface Area of Montmorillonite samples. **Supporting Table S2:** The thickness of the diffuse layer at various Al^3+^ concentrations (*κ* ^−1^/nm).

## Author Contributions

**Hanyu Zhao**: data curation (equal), formal analysis (equal), investigation (equal), resources (equal), software (equal), validation (equal), visualization (equal), writing – original draft (equal). **Lijun Liu**: conceptualization (equal), funding acquisition (equal), methodology (equal), project administration (equal), supervision (equal), writing – review & editing (equal). **Xue Yuan**: data curation (equal), investigation (equal), resources (equal). **Qingtao Pang**: funding acquisition (equal), methodology (equal), project administration (equal), supervision (equal). **Lei Yao**: conceptualization (equal), funding acquisition (equal), methodology (equal), project administration (equal). **Bo Gao**: data curation (equal), formal analysis (equal), investigation (equal), resources (equal), software (equal), validation (equal), visualization (equal). **Yihong Li**: formal analysis (equal), writing – review & editing (equal). **Liang Han**: formal analysis (equal), validation (equal), visualization (equal). **Yizhe Yang**: formal analysis (equal), validation (equal). **W** **ei Yu**: data curation (equal), investigation (equal), resources (equal). **Zhen Li**: writing – review & editing (equal). **Yuexian Yu**: formal analysis (equal), validation (equal). **Jinzhou Qu**: methodology (equal).

## Funding

This work was supported by the Natural Science Basic Research Plan in Shaanxi Province of China (Grant 2021JLM‐15), High‐level Innovation and Entrepreneurship Talent Project of Department of Science and Technology of Shaanxi Province (Grant QCYRCXM‐2023‐054), Natural Science Basic Research Program of Shaanxi Province (Grant 2025JC‐YBMS‐398).

## Conflicts of Interest

The authors declare no conflicts of interest.

## Supporting information

Supplementary Material

## Data Availability

The data that support the findings of this study are available from the corresponding author upon reasonable request.
