# Supplementary material for: Impact of Al3+ Concentration on Montmorillonite Sedimentation: Insights Into Particle Size Behavior
Source: ChemistryOpen. 2025 Dec 3;15(4):e202500446. doi: 10.1002/open.202500446 (PMC13052313; doi:10.1002/open.202500446)
Supplement: Supplementary file 1 — Supplementary Material [file OPEN-15-e202500446-s001.pdf]

Supporting Information files

1.Supplementary Figure

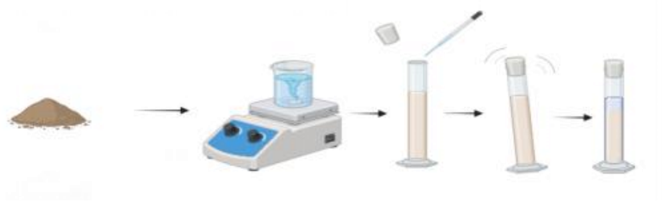

Figure 1. Schematic representation of the montmorillonite sedimentation process.

Experimental Procedure:

Montmorillonite (2.5 g) was added to a beaker containing deionized water and stirred at 500 r/min for 30 min using a magnetic stirrer to achieve a homogeneous dispersion, resulting in a uniform 10 g/L slurry. Al^{3+} solutions with concentrations of 10, 15, 20, 25, and 30 mmol/L were prepared in advance. Various amounts of the Al^{3+} solutions were introduced into the suspension. The mixture was then carefully transferred to a 250 mL graduated cylinder, which was inverted 10 times to ensure thorough mixing. Immediately after inversion, the cylinder was placed upright on the laboratory bench, and the sedimentation process of the suspension was observed.

List of Experimental Equipment

Instrument Name	Model	Manufacturer
X-ray Diffractometer	Bruker D8 ADVANCE A25 X	Bruker, Germany
Fourier Transform Infrared Spectrometer	Nicolet 6700	Thermo Fisher Scientific, USA
Zeta Potential and Nanoparticle Size Analyzer	Malvern ZEN3690	Malvern Panalytical, UK
Turbidimeter	WZS-188	Shanghai Leici Instrument Co., Ltd., China
Polarizing Microscope	ZEISS Stemi 508	ZEISS, Germany
Laser Diffraction Particle Size Analyzer	LS13 320	Beckman Coulter, USA
Scanning Electron Microscope	ZEISS GeminiSEM 360	ZEISS, Germany

Figures 2 to 4 demonstrate the distinct characteristics of the two samples, with sample1 defined as having a coarser d50 particle size and sample 2 as having a finer d50 particle size.

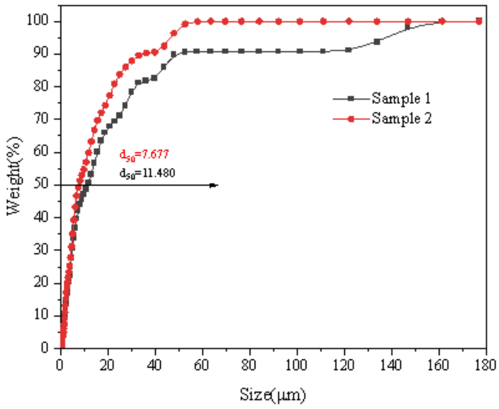

Figure 2. Particle size analysis of montmorillonite samples.

Particle size distribution data of the samples

Diameter (Lower)/um	Diff. Volume %	Diameter (Upper)/um	Cum. Volume %	Diameter (Lower)/um	Diff. Volume %	Diameter (Upper)/um	Cum. Volume %
0.044	0	0.04	0	0.044	0	0.04	0
0.048	0	0.044	0	0.048	0	0.044	0
0.053	0	0.048	0	0.053	0	0.048	0
0.058	0	0.053	0	0.058	0	0.053	0
0.064	0	0.058	0	0.064	0	0.058	0
0.07	0	0.064	0	0.07	0	0.064	0
0.077	0	0.07	0	0.077	0	0.07	0
0.084	0	0.077	0	0.084	0	0.077	0
0.093	0	0.084	0	0.093	0	0.084	0
0.102	0	0.093	0	0.102	0	0.093	0
0.112	0	0.102	0	0.112	0	0.102	0
0.123	0	0.112	0	0.123	0	0.112	0
0.134	0	0.123	0	0.134	0	0.123	0
0.148	0	0.134	0	0.148	0	0.134	0
0.162	0	0.148	0	0.162	8.3E-5	0.148	0
0.178	0	0.162	8.3E-5	0.178	0.0014	0.162	0
0.195	2.2E-4	0.178	0.0015	0.195	0.0059	0.178	0
0.214	0.0048	0.195	0.0073	0.214	0.014	0.195	4.5E-4
0.235	0.025	0.214	0.022	0.235	0.027	0.214	0.0081
0.258	0.068	0.235	0.049	0.258	0.046	0.235	0.041
0.284	0.13	0.258	0.095	0.284	0.067	0.258	0.12
0.311	0.21	0.284	0.16	0.311	0.09	0.284	0.27
0.342	0.29	0.311	0.25	0.342	0.11	0.311	0.49
0.375	0.36	0.342	0.36	0.375	0.13	0.342	0.8
0.412	0.42	0.375	0.49	0.412	0.15	0.375	1.17
0.452	0.45	0.412	0.64	0.452	0.16	0.412	1.59
0.496	0.45	0.452	0.8	0.496	0.17	0.452	2.03

0.545	0.42	0.496	0.96	0.545	0.17	0.496	2.47
0.598	0.36	0.545	1.13	0.598	0.18	0.545	2.88
0.657	0.28	0.598	1.31	0.657	0.19	0.598	3.23
0.721	0.19	0.657	1.5	0.721	0.21	0.657	3.5
0.791	0.11	0.721	1.7	0.791	0.24	0.721	3.67
0.869	0.072	0.791	1.94	0.869	0.29	0.791	3.77
0.954	0.083	0.869	2.23	0.954	0.37	0.869	3.84
1.047	0.17	0.954	2.6	1.047	0.48	0.954	3.93
1.149	0.35	1.047	3.08	1.149	0.62	1.047	4.12
1.261	0.62	1.149	3.7	1.261	0.78	1.149	4.49
1.385	0.95	1.261	4.49	1.385	0.96	1.261	5.14
1.52	1.31	1.385	5.45	1.52	1.14	1.385	6.14
1.669	1.64	1.52	6.58	1.669	1.29	1.52	7.5
1.832	1.89	1.669	7.87	1.832	1.42	1.669	9.2
2.011	2.02	1.832	9.29	2.011	1.5	1.832	11.2
2.208	2	2.011	10.8	2.208	1.54	2.011	13.2
2.423	1.84	2.208	12.3	2.423	1.55	2.208	15.3
2.66	1.61	2.423	13.9	2.66	1.55	2.423	17.2
2.92	1.39	2.66	15.4	2.92	1.57	2.66	18.9
3.206	1.3	2.92	17	3.206	1.65	2.92	20.4
3.519	1.43	3.206	18.7	3.519	1.81	3.206	21.8
3.863	1.83	3.519	20.5	3.863	2.07	3.519	23.4
4.241	2.46	3.863	22.5	4.241	2.4	3.863	25.3
4.656	3.18	4.241	24.9	4.656	2.75	4.241	27.9
5.111	3.76	4.656	27.7	5.111	3.02	4.656	31.2
5.611	4	5.111	30.7	5.611	3.15	5.111	35.1
6.159	3.81	5.611	33.9	6.159	3.07	5.611	39.3
6.761	3.26	6.159	36.9	6.761	2.79	6.159	43.3
7.422	2.56	6.761	39.7	7.422	2.37	6.761	46.7
8.148	2.01	7.422	42.1	8.148	1.93	7.422	49.3
8.944	1.8	8.148	44	8.944	1.59	8.148	51.3
9.819	1.99	8.944	45.6	9.819	1.46	8.944	53
10.78	2.42	9.819	47.1	10.78	1.6	9.819	54.8
11.83	2.85	10.78	48.7	11.83	2.01	10.78	57
12.99	3.15	11.83	50.7	12.99	2.63	11.83	59.9
14.26	3.22	12.99	53.3	14.26	3.26	12.99	63.3
15.65	2.87	14.26	56.6	15.65	3.55	14.26	66.8
17.18	2.32	15.65	60.1	17.18	3.28	15.65	69.8
18.86	2.28	17.18	63.4	18.86	2.59	17.18	72.1
20.71	2.93	18.86	66	20.71	1.89	18.86	74.3
22.73	3.4	20.71	67.9	22.73	1.55	20.71	77.4
24.95	2.74	22.73	69.4	24.95	1.81	22.73	80.9
27.39	1.79	24.95	71.2	27.39	2.97	24.95	83.8
30.07	1.78	27.39	74.2	30.07	4.35	27.39	86

33.01	2.51	30.07	78.5	33.01	2.63	30.07	87.9
36.24	2.16	33.01	81.2	36.24	0.52	33.01	89.6
39.78	1.1	36.24	81.7	39.78	0.94	36.24	90.3
43.67	1.36	39.78	82.6	43.67	3.52	39.78	90.6
47.94	3.09	43.67	86.1	47.94	3.55	43.67	92.4
52.63	3.49	47.94	89.7	52.63	1.05	47.94	96.4
57.77	1.36	52.63	90.7	57.77	0.06	52.63	99.2
63.42	0.1	57.77	90.8	63.42	0	57.77	99.96
69.62	0	63.42	90.8	69.62	0	63.42	100
76.43	0	69.62	90.8	76.43	0	69.62	100
83.9	0	76.43	90.8	83.9	0	76.43	100
92.1	0	83.9	90.8	92.1	0	83.9	100
101.1	0	92.1	90.8	101.1	0	92.1	100
111	0	101.1	90.8	111	0	101.1	100
121.8	0	111	90.8	121.8	0.26	111	100
133.7	0	121.8	91.1	133.7	2.65	121.8	100
146.8	0	133.7	93.7	146.8	4.39	133.7	100
161.2	0	146.8	98.1	161.2	1.77	146.8	100
176.9	0	161.2	99.9	176.9	0.13	161.2	100
194.2	0	176.9	100	194.2	0	176.9	100

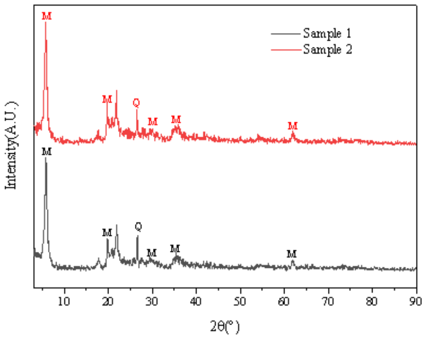

Figure 3. X-ray diffraction analysis of montmorillonite samples.

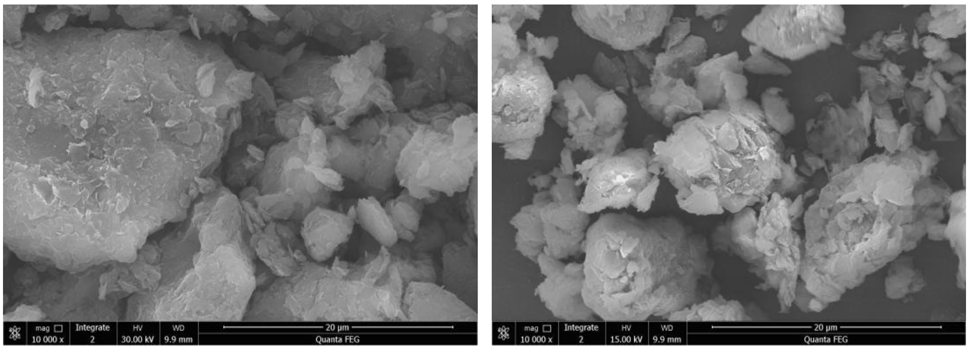

(a) (b)

Figure 4. SEM of montmorillonite samples. (a) Sample 1; (b) Sample 2

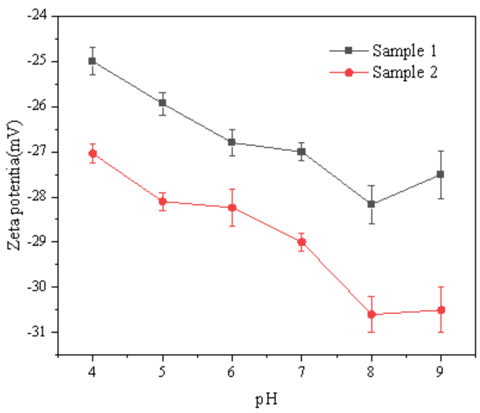

Figure 5. Zeta potential analysis of montmorillonite samples.

Surface zeta potential data of the samples

pH	Sample 1			RDS	Sample 2			RDS
4	-24.7	-25.0	-25.3	0.01	-27.2	-26.8	-27.1	0.01
5	-25.9	-25.7	-26.2	0.01	-28.1	-27.9	-28.3	0.01
6	-26.5	-27.1	-26.8	0.01	-28.1	-28.7	-27.9	0.01
7	-27.0	-27.2	-26.8	0.01	-29.2	-29.0	-28.8	0.01
8	-27.7	-28.3	-28.5	0.01	-30.6	-30.2	-31.0	0.01
9	-26.9	-27.9	-27.7	0.02	-30.5	-30.0	-31.0	0.02

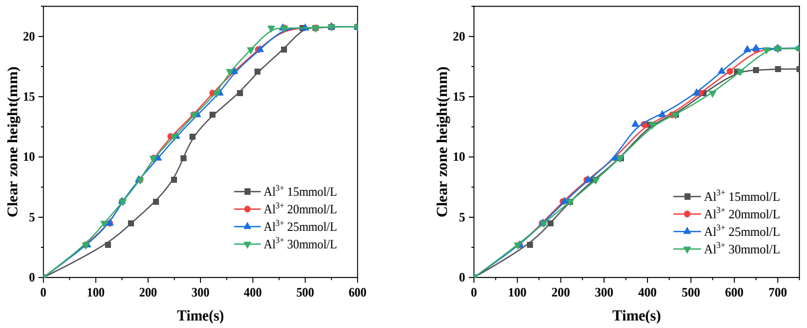

(a) (b)

Figure 6. Evolution of the settling height for the understudied montmorillonite samples. (a) Sample 1; (b) Sample 2

Temporal changes in supernatant height during sedimentation

Sample 1

Al^{3+} concentrations 15 mmol/L		Al^{3+} concentrations 20 mmol/L		Al^{3+} concentrations 25 mmol/L		Al^{3+} concentrations 30 mmol/L	
Time（s）	Hight/mm	Time（s）	Hight/mm	Time（s）	Hight/mm	Time（s）	Hight/mm
0	0	0	0	0	0	0	0
123	2.7	81	2.7	84	2.7	81	2.7
167	4.5	127	4.5	127	4.5	116	4.5
215	6.3	150	6.3	150	6.3	152	6.3
249	8.1	185	8.1	182	8.1	185	8.1
267	9.9	211	9.9	219	9.9	210	9.9
285	11.7	243	11.7	254	11.7	250	11.7
323	13.5	287	13.5	294	13.5	288	13.5
375	15.3	323	15.3	337	15.3	331	15.3
409	17.1	364	17.1	365	17.1	356	17.1
459	18.9	410	18.9	414	18.9	396	18.9
495	20.7	460	20.7	457	20.7	435	20.7
520	20.7	520	20.7	500	20.7	460	20.7
550	20.8	550	20.8	550	20.8	520	20.7
600	20.8	600	20.8	600	20.8	550	20.8
						600	20.8

Sample 2

Al^{3+} concentrations 15 mmol/L		Al^{3+} concentrations 20 mmol/L		Al^{3+} concentrations 25mmol/L		Al^{3+} concentrations 30mmol/L	
Time（s）	Hight/mm	Time（s）	Hight/mm	Time（s）	Hight/mm	Time（s）	Hight/mm
0	0	0	0	0	0	0	0
129	2.7	105	2.7	106	2.7	101	2.7
176	4.5	157	4.5	159	4.5	161	4.5
221	6.3	205	6.3	209	6.3	222	6.3
275	8.1	260	8.1	263	8.1	281	8.1
339	9.9	328	9.9	327	9.9	337	9.9
403	12.7	392	12.7	372	12.7	412	12.7
465	13.5	457	13.5	434	13.5	465	13.5
529	15.3	521	15.3	513	15.3	550	15.3
606	17.1	590	17.1	571	17.1	614	17.1
650	17.2	650	18.9	630	18.9	674	18.9
700	17.3	700	19	650	19	700	19
750	17.3	750	19	700	19	750	19

750	19.05

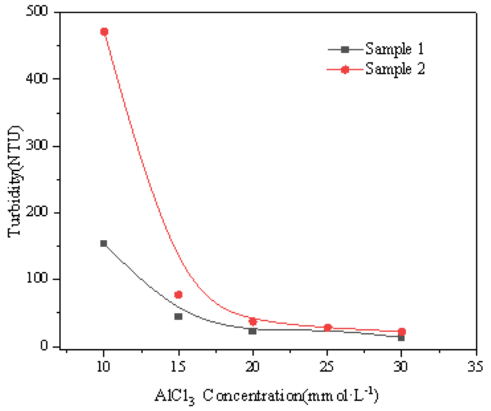

Figure 7. Supernatant turbidity in terms of the $AlCl_3$ concentration for both samples.

Turbidity measurement data

Al^{3+} concentrations/mmol^{-1}	Sample 1			RDS	Sample 2			RDS
10	156.70	153.20	145.50	0.04	471.10	468.50	470.10	0.01
15	44.55	45.06	44.97	0.01	77.25	75.89	77.20	0.01
20	22.36	23.74	23.02	0.03	37.26	36.78	36.32	0.01
25	26.27	25.45	25.89	0.02	28.30	27.27	28.60	0.02
30	13.40	14.29	13.60	0.03	22.35	22.36	21.79	0.01

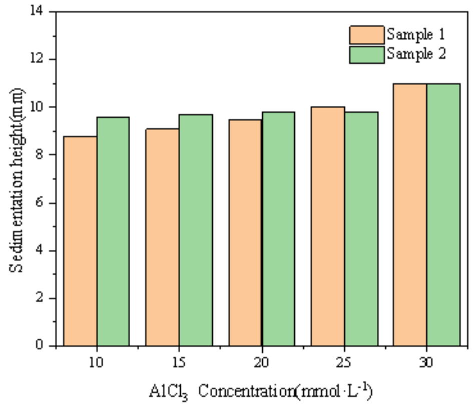

Figure 8. Sediment layer heights of both samples for various concentrations of $AlCl_3$.

Sediment bed height data

Al^{3+} concentrations/mmol^{-1}	Sample 1			RDS	Sample 2			RDS
10	8.8	8.7	8.8	0.01	9.6	9.5	9.8	0.02
15	9.1	9.3	9.0	0.02	9.7	9.9	9.5	0.02
20	9.5	9.7	9.9	0.02	9.8	10.0	9.5	0.03

25	10	10.3	10.1	0.02	9.8	10.1	9.6	0.03
30	11	10.8	11.1	0.01	11	10.7	11.1	0.02

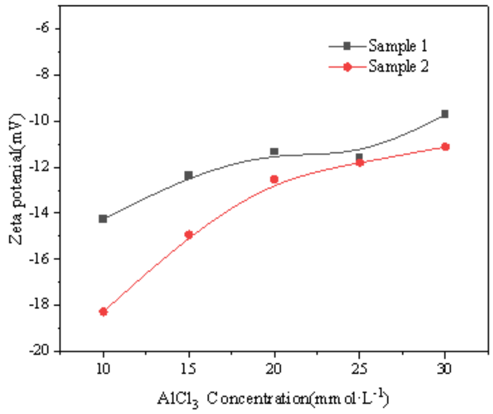

Figure 9. Effect of the Al^{3+} concentrations on the Zeta potential of the understudied montmorillonite samples.

Surface zeta potential data after sedimentation

Al^{3+} concentrations/$mmol^{-1}$	Sample 1			RDS	Sample 2			RDS
10	-14.8	-14.2	-14.1	0.02	-18.5	-18.2	-18.1	0.01
15	-12.6	-11.9	-12.9	0.04	-14.7	-15.5	-14.6	0.03
20	-11.9	-11.2	-11.3	0.03	-12.3	-13.3	-12	0.05
25	-12.9	-12.7	-12.1	0.03	-11.4	-12.1	-11.9	0.03
30	-8.59	-9.24	-9.07	0.04	-11.1	-10.78	-11.48	0.03

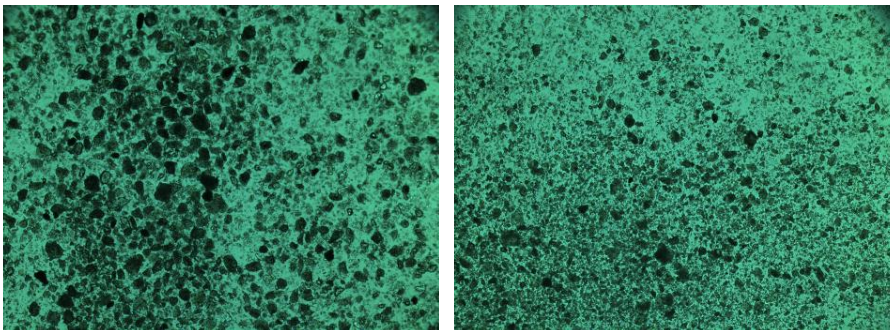

(a) (b)

Figure 10. Micro-morphology of flocs in the understudied montmorillonite samples under the action of Al^{3+} ions. (a) Sample 1; (b) Sample 2

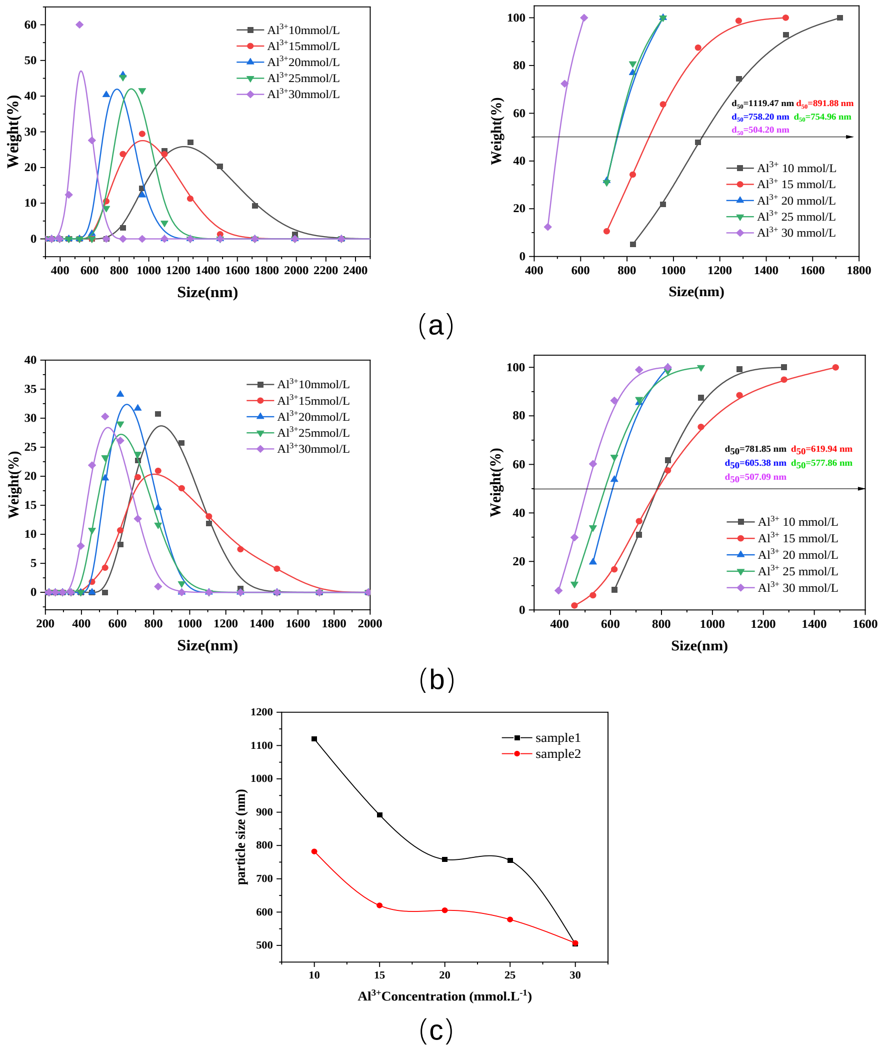

Figure 11. Particle size distribution of the supernatant for both samples after sedimentation of montmorillonite: (a) Sample 1; (b) Sample 2; (c) Plots of the particle size in terms of the concentration.

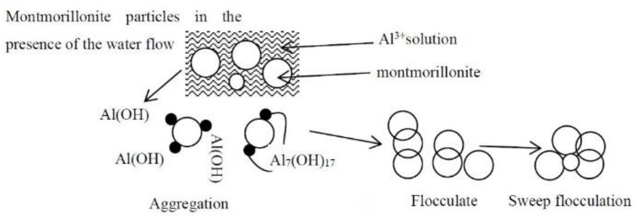

Figure 12. Mechanism of aggregation and sedimentation by Al^{3+}.

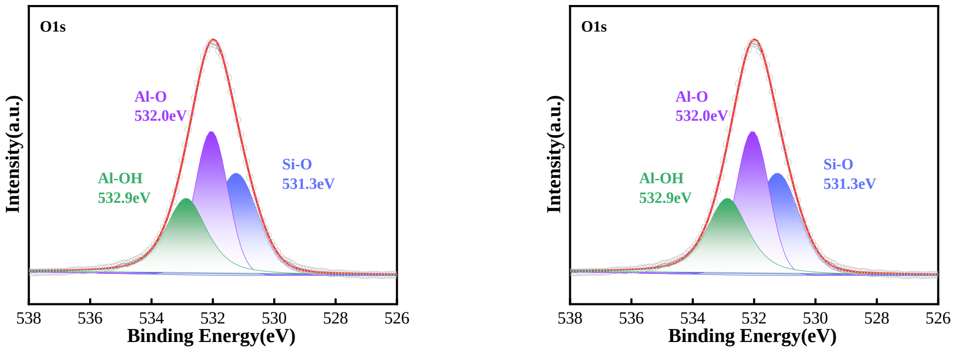

(a)

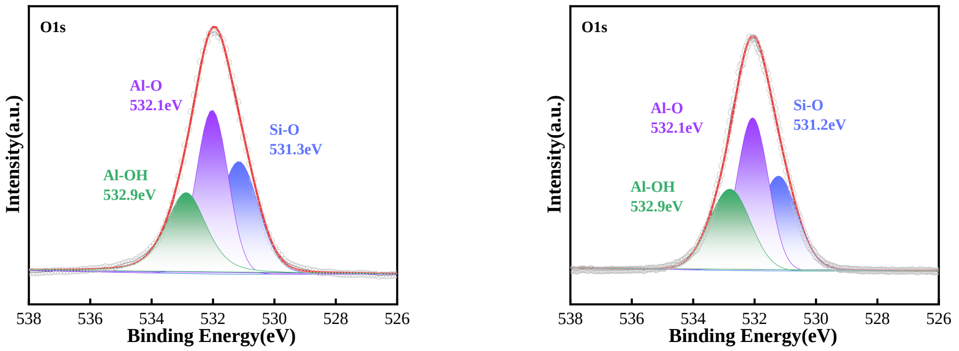

(b)

Supplementary Figure 13. XPS spectra of montmorillonite at various Al^{3+} concentrations: (a) Pristine; (b) After sedimentation.

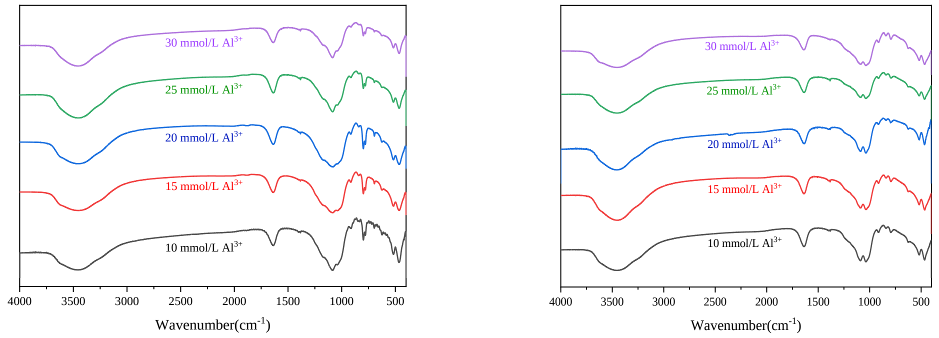

(a) (b)

Supplementary Figure 14. FTIR spectra of montmorillonite at various Al^{3+} concentrations. (a) Sample 1; (b) Sample 2

2 The raw data for tables

The raw data are available in SI Table 1

Table 1. BET Surface Area of Montmorillonite samples

	Specific surface area（m^2/g）
the sample of coarse particles	48.5517
the sample of fine particles	52.9531

The raw data are available in SI Table 2

For an aqueous solution at 25°C: $\kappa = 3.29 \times 10^9 (\sum c_i Z_i^2)^{1/2} m^{-1}$ (where κ represents the Debye constant, and ci and Zi in order are the amount-of-substance concentration and the charge number of ions).

Table 2. The thickness of the diffuse layer at various Al^{3+} concentrations (κ^{-1}/nm)

Al^{3+} Concentration (mol/L)	2×10^{-4}	4×10^{-4}	6×10^{-4}	8×10^{-4}	10×10^{-4}	12×10^{-4}
κ^{-1}/nm	7.16	5.06	4.14	3.58	3.20	2.92

The size of κ $^{-1}$ affects the speed of the potential decrease with distance, and can effectively control the range of electrostatic force b

Formula derivation process

$$K = \frac{\pi g \Delta\rho d^3 H(1-\varphi_F)}{12kT\ln(1/\varphi_F)} \quad (1)$$

In order to evaluate the change of entropy in the solid-liquid separation system of flocs during the separation process, some idealized assumptions need to be introduced. First of all, it is necessary to assume that the properties of flocs are homogeneous, that is, the particle size, shape, density and so on are consistent. If flocs are assumed to be rigid particles, the thermodynamic equations previously established for solid-liquid separation systems can be applied to study floc separation. Therefore, thermodynamic parameters such as entropy and free energy can be utilized in floc separation to determine the following indicators: ① Establish a criterion for evaluating floc separation efficiency; ② Propose a feasibility criterion to determine the viability of the floc separation process; ③ Provide a rough estimate of the void fraction in the sediment. Furthermore, there is no change in thermodynamic internal energy when it exchanges spatial positions with an equal volume of water. The bulk particles are regarded as molecules in a chemical system. The possible number of arrangements (or multiplicity) of these flocculent particles and water particles is

$$W = (N_1 + N_2)!/(N_1!N_2!) \quad (1)$$

In the formula, N_1 and N_2 represent the number of water level points for the remaining volume of the particle, respectively.

According to molecular thermodynamics, the entropy S of this system is

$$S = k\ln W \quad (2)$$

in the formula： $k = R/L$ ——boltzmann constant ， $k = 1.38\times10^{-23}$ J/K；

R——gas constant ， R=8.314/k•mol；

L——Avogadro's number ， $L = 6.02\times10^{23}$ mol。

The entropy of solid-liquid suspension systems can be obtained by combining the Stirling approximation formula (1) with equation (2).

$$S_{SL} = -k'\left[N_1\ln\frac{N_1}{N_1+N_2} + N_2\ln\frac{N_2}{N_1+N_2}\right] \quad (3)$$

The above equation can be rewritten as:

$$S_{SL}=-\frac{N_1}{N_1+N_2}\ln\frac{N_1}{N_1+N_2}-\frac{N_2}{N_1+N_2}\ln\frac{N_2}{N_1+N_2} \quad (4)$$

in the formula，$S_{SL}=S_{SL}/k'（N_1+N_2）$ Can be defined as the specific entropy of a suspension per unit volume.

in equation (4) $\frac{N_1}{N_1+N_2}=\varphi_F$， The volume concentration of floc particles in the feed system, so the equation can be written as:

$$S_{SL}=-\varphi_F\ln\varphi_F-(1-\varphi_F)\ln(1-\varphi_F) \quad (5)$$

In an ideal separation process, the solid-liquid separation of flocs should completely separate them into pure flocs and water, with the system's entropy reaching zero. This assumes an initial volume concentration φ_F of floc ions in the system, which becomes φ_U =1 in the underflow after separation, while the overflow contains no floc particles (volume concentration φ_0 = 0). However, in practice, complete separation is impossible. Pure flocs and water are rarely obtained, as the underflow or filter cake always contains residual water, while the overflow or filtrate retains fine floc particles. The separation efficiency can be evaluated using the entropy index.

If the entropy of the initial system is defined as 100%, the reduction in entropy after separation can be regarded as the separation efficiency of the system. In this case, the separation efficiency is defined as the entropy index E_s, which is mathematically expressed as:

$$E_S=[S_F-(S_U+S_o)]/S_F \quad (6)$$

$$E_S=[Q_FS_F-Q_US_U-(Q_F-Q_U)S_O)]/Q_FS_F$$

In equation (6), the entropy S denotes the total entropy of the fluid. When using the specific entropy S per unit volume of suspension, the equation (6) should be

$$E_S=[Q_FS_F-Q_US_U-(Q_F-Q_U)S_O)]/Q_FS_F$$

where Q is the volumetric flow rate of the suspension, with subscripts F, U, and O denoting feed, underflow, and overflow, respectively.

或$E_S = [S_F - \frac{Q_U S_U}{Q_F} - (1 - \frac{Q_U}{Q_F}) S_O)] / S_F$ （7）

or $E_S = [S_F - \frac{Q_U S_U}{Q_F} - (1 - \frac{Q_U}{Q_F}) S_O)] / S_F$ （7）

Equation (7) can be used as a basic evaluation method for the solid-liquid separation efficiency of flocs. However, in practice, the task of flocculation clarification is to obtain clarified liquid, while the purity of the bottom flow flocs is secondary.

Based on the above flocculation solid-liquid separation efficiency, the change of system entropy, and the change of internal energy, a criterion similar to the free energy in chemical reaction can be derived from the thermodynamic relationship to explain whether the solid-liquid separation system process can be carried out spontaneously.

According to chemical thermodynamics, the changes of the work function ΔG , internal energy ΔU , and entropy ΔS can be calculated for a non-flowing system under isothermal and isochoric conditions as follows

$\Delta G = \Delta U - T\Delta S$ （8）

where T is the absolute temperature.

For a spontaneous process, the work function decreases, i.e., the value is negative. Typically, for an automatic process, both the ΔU and ΔS is also negative values. In this case, it should be

$-T\Delta S < -\Delta U$ （9）

Therefore, the solid-liquid separation criterion of flocs can be defined as

$K = \Delta U / T\Delta S$ （10）

The separation process can be performed automatically when $K > 1$and cannot be performed when $K < 1$.

For equation (10), the key issue is how to calculate the change in internal energy ΔU and entropy ΔS of a flocculent solid-liquid separation system. Some attempts have been made, but only for relatively simple solid-liquid separation systems, such as gravity sedimentation.

When calculating entropy, certain assumptions must be made: (1) The flocculation system is a homogeneous particle dispersion system with uniform particle size and consistent sphericity coefficient; (2) The solid-liquid system of flocs is completely separated, resulting in pure flocs

and pure water.

(3) The so-called "rule solution" is used, that is, there is no change in the mixing free energy when the flocculant particles are mixed with water molecules; (4) the initial suspension is a dilute suspension.

The system's entropy can be written as

$$\Delta S = -kN \ln(V_0 / V_U) \quad (11)$$

where k is the Boltzmann constant, N is the number of floc particles in the system, V_0 is the volume of the upper clear night, and V_U is the volume occupied by the flocs.

For the free monomer particles, the change of internal energy is the change of potential energy in the system. In the process of sedimentation separation, the change of potential energy of particles is:

$$\Delta U = -NV_p \Delta \rho g (H/2 - h/2) \quad (12)$$

$$\Delta U = -NV_p \Delta \rho g (H/2 - h/2) \quad (12)$$

in the formula Vp——Volume of a single particle, $V_P = \pi d^3 / 6$;

$\Delta \rho$ ——Solid-liquid density difference, $\Delta \rho = \rho_S - \rho_L$;

g——acceleration of gravity ;

H——Height of floc suspension before sedimentation;

h——Settled floc suspension height.

The following separation criteria can be obtained from (10) to (12):

$$K = \frac{\pi g \Delta \rho d^3 H (1 - \varphi_F)}{12 kT \ln(1/\varphi_F)} \quad (13)$$

As shown in Equation (13), the magnitude of the separation criterion is largely determined by the sedimentation height H and particle diameter.

Assuming the feed temperature is 20℃, the volume concentration of flocculation is 5% and 1%, the sedimentation height is 20cm, $\Delta \rho$ =1600kg/m^3, and the particle size of separation is

1μm and 0.013μm, the separation criterion is calculated.

Solution：given g=9.80m/s^2，d=10^{-6}m，k=1.38×10^{-23}J/K，Substitute the above data into the formula（13），obtain $K = 2.03\times10^5(1-\varphi_F)/(\ln(1/\varphi_F)$。

when $\varphi_F = 0.05$， $(1-\varphi_F)/(\ln(1/\varphi_F) = 0.317$， then K=64.375；

when $\varphi_F = 0.01$， $(1-\varphi_F)/(\ln(1/\varphi_F) = 0.215$， then K=43.640；

When d=0.013μm，namely d=1.3×10^{-8}m,

When $\varphi_F = 0.05$，then K=0.1416；

When $\varphi_F = 0.01$，then K=0.096；

As observed, when d=1μm, the separation criterion K exceeds 1 at feed floc volume concentrations of 5% and 1%, enabling automatic separation. However, when particle size decreases to 0.013μm, K drops below 1, falling below the critical point for sedimentation separation, thereby preventing automatic separation.

Funding support

This work was supported by the Natural Science Basic Research Plan in Shaanxi Province of China[grant number 2025JC-YBMS-398 and grant number 2021JLM-15]; and Key R&D Program of Shaanxi Province(2024CY2-GJHX-69)is gratefully acknowledged.
